# Kaposiform Hemangioendothelioma of the Oral Cavity in an Adult Woman: A Case Report

**DOI:** 10.3390/jcm14228228

**Published:** 2025-11-20

**Authors:** Martina Caputo, Gaspare Palaia, Daniele Pergolini, Alessandra Putrino, Amelia Bellisario, Gianluca Tenore, Federica Rocchetti, Angela Galeotti, Cira Rosaria Tiziana Di Gioia, Umberto Romeo

**Affiliations:** 1 Dentistry Unit, Management Innovations, Diagnostics and Clinical Pathways, Bambino Gesù Children’s Hospital, IRCCS, 00165 Rome, Italy; 2 Department of Oral Sciences and Maxillofacial Surgery, Sapienza University of Rome, 00161 Rome, Italygianluca.tenore@uniroma1.it (G.T.); federica.rocchetti@uniroma1.it (F.R.);; 3 U.N.-E.U. International Research Project on Human Health, Oral Health Section, 1200 Geneva, Switzerland; 4 Department of Radiology, Oncology and Pathology, Sapienza University of Rome, 00161 Rome, Italy

**Keywords:** vascular tumors, kaposiform hemangioendothelioma, Kasabach–Merritt Syndrome

## Abstract

**Background:** Kaposiform hemangioendothelioma (KHE) is a rare, locally aggressive vascular tumor that shares histological features with Kaposi’s sarcoma. It usually occurs in infancy or early childhood and is seldom reported in adults. The most common sites are the skin and retroperitoneum, whereas the head, neck, and mediastinum are less frequently involved. KHE rarely regresses spontaneously, and metastasis is uncommon, but up to 70% of cases may develop Kasabach–Merritt Syndrome (KMS), a life-threatening coagulopathy. Here, we present an unusual case of KHE in an adult patient, emphasizing the importance of early recognition and management. **Methods:** A 39-year-old woman with systemic lupus erythematosus presented with an exophytic lesion in the left retromolar region. Clinical and radiological evaluations were followed by both incisional and excisional biopsies. Histopathological and immunohistochemical analyses were performed, and surgical resection with wide margins was undertaken according to recommendations from a multidisciplinary tumor board. **Results:** Histology revealed spindle cell clusters, slit-like vascular spaces, endothelial cells with eosinophilic cytoplasm, and immunopositivity for CD31, CD34, and smooth muscle actin, confirming the diagnosis of KHE. Given the tumor’s locally aggressive behavior and potential risk of KMS, extended surgical excision was performed. **Conclusions:** This case underscores the diagnostic challenges of KHE in adults and highlights the essential role of histopathology, immunohistochemistry, and multidisciplinary evaluation. Prompt diagnosis and radical surgical management are critical to preventing complications and improving patient outcomes.

## 1. Introduction

Kaposiform hemangioendothelioma (KHE) is a rare, locally aggressive vascular neoplasm with histopathological similarities to Kaposi’s sarcoma [1]. It predominantly arises in infancy or early childhood, and only a small number of adult cases have been described [2]. Establishing its true incidence is challenging, as atypical or asymptomatic lesions may be misdiagnosed as variants of infantile hemangioma (IH) or other vascular anomalies. In one North American study, the annual incidence in children was estimated at approximately 1 in 1,400,000 [3], with nearly 90% of cases diagnosed during the first year of life.

KHE most commonly affects the skin and retroperitoneum, while involvement of the head and neck, soft tissues of the extremities, trunk, or mediastinum is less frequent [4,5]. Although histologically benign, the tumor may cause severe morbidity, and in up to 70% of patients it is complicated by Kasabach–Merritt Syndrome (KMS), a consumptive coagulopathy associated with high mortality [6].

The entity was first described by Zukerberg et al. in 1993 as distinct from IH due to its locally infiltrative growth, aggressive clinical course, and “Kaposi-like” histological appearance [7]. Superficial lesions typically present as firm, violaceous, and painful masses, while deeper lesions—arising in the retroperitoneum, thoracic cavity, or muscle—may remain clinically silent, resulting in delayed diagnosis. Regional lymph node involvement has been observed, although distant metastasis has not been reported [8].

Unlike Kaposi’s sarcoma, which is linked to human herpesvirus 8, the pathogenesis of KHE remains uncertain. Evidence suggests a possible lymphatic origin, supported by the expression of both vascular (CD31, CD34, VEGFR-3) and lymphatic markers (D2-40, PROX1) in spindle cells [9]. While most cases occur without an obvious trigger, trauma or infection may contribute to symptom progression [10]. A slight male predominance has been described, and approximately half of all cutaneous lesions are present at birth [11,12]. In contrast to juvenile hemangiomas, KHE rarely undergoes spontaneous regression.

The pathophysiology of KMS is not fully understood. Current hypotheses suggest that abnormal platelet activation and sequestration within the tumor result in the consumption of clotting factors [13,14]. Other studies propose that the abnormal vascular architecture of KHE may generate turbulent blood flow and platelet trapping [5]. Clinically, KMS is refractory to platelet transfusions and carries a significant risk of life-threatening hemorrhage [15].

Histologically, KHE is characterized by spindle cell clusters with minimal atypia, slit-like vascular spaces, microthrombi, and hemosiderin deposits [1]. Treatment strategies vary and include surgical excision, embolization, compression, laser therapy, radiotherapy, and systemic therapy. Radical excision, when feasible, remains the most effective treatment. Medical therapies—including vincristine, corticosteroids, propranolol, interferon, and sirolimus—have been used with variable outcomes, and no universally accepted protocol exists. Prognosis largely depends on lesion size, anatomical site, and association with KMS [16].

## 2. Case Report

**Figure 1:**
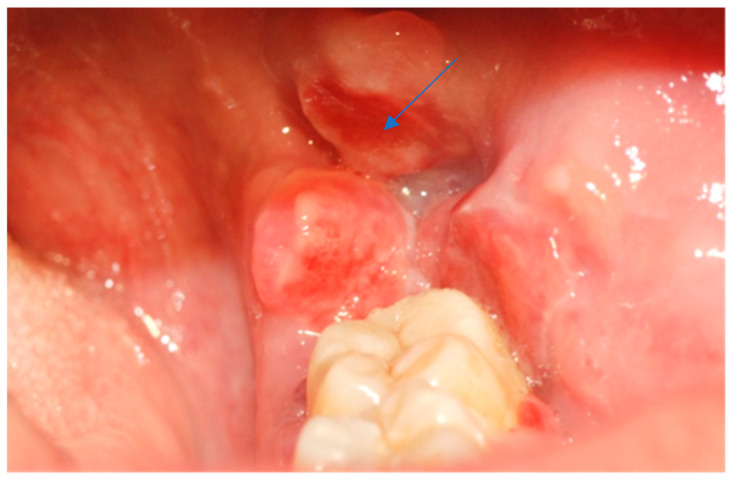
Erythematous and exophytic lesion located on the left retromolar trigone. The blue arrow indicates the lesion located at the retromolar trigone.

A 39-year-old Caucasian woman was referred by her dentist to the Department of Oral Sciences and Maxillofacial Surgery, Polyclinic Umberto I, “Sapienza” University of Rome, for evaluation of multiple exophytic and erythematous lesions in the left retromolar region (Figure 1). The lesions had been present for approximately five months and were associated with occasional minor bleeding following local trauma. The patient also reported mild pain and a recent increase in the size of the lesions during the two weeks preceding the visit. Her medical history included systemic lupus erythematosus (SLE), diagnosed in 2000 at the Department of Clinical Medicine, “Sapienza” University of Rome, following symptoms of gastroesophageal reflux, low-grade fever, diffuse arthromyalgia, a previous pericardial effusion, and positivity for ANA and ENA SS-A antibodies. She was under treatment with prednisone and cyclophosphamide, levothyroxine sodium for hypothyroidism, and omeprazole for gastroesophageal reflux. The patient was allergic to penicillins and denied regular tobacco or alcohol use. In 2015, she developed ocular vasculitis with photosensitivity, prompting replacement of cyclosporine with cyclophosphamide.

**Figure 2:**
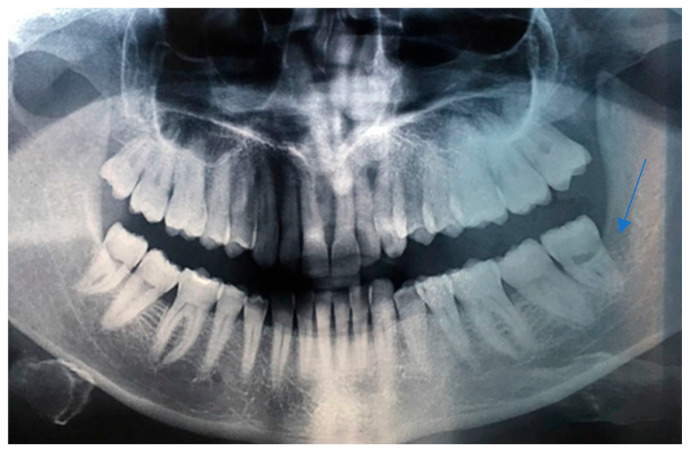
OPG x-ray and the blue arrow show a focus of radiolucency behind the lower-left third molar.

**Figure 3:**
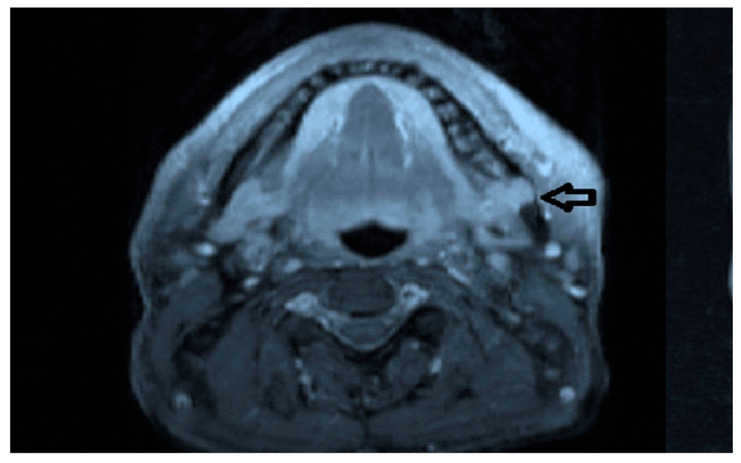
MRI view of the left retromolar region showing with the indication of black arrow a soft-tissue lesion corresponding to the clinical site of the Kaposiform hemangioendothelioma.

**Figure 4:**
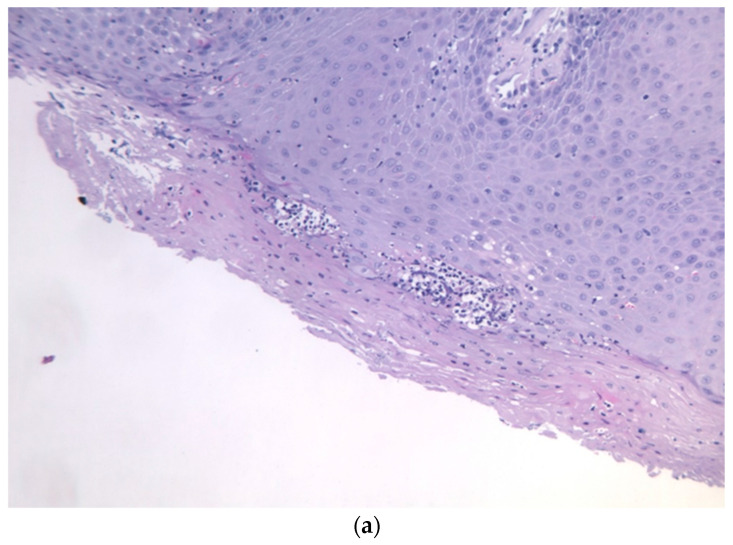

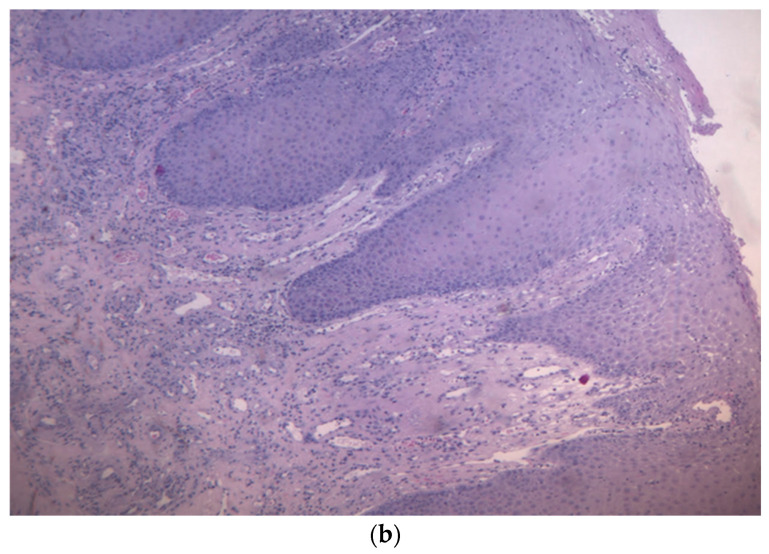
(**a**,**b**): Histological section with evidence of malpighian epithelium with mild hyperorthokeratosis.

Intraoral examination revealed an exophytic erythematous lesion measuring approximately 2.5 × 2 cm on the left retromolar trigone, while orthopantomographic X-ray imaging demonstrated a corresponding radiolucency in the same region (Figure 2). Considering her systemic condition and ongoing therapy with cyclophosphamide, a multidisciplinary management plan was initiated, which included second-level imaging (MRI) to evaluate lesion extension (Figure 3), a professional oral hygiene session, chlorhexidine 0.12% mouth rinses, and selective grinding of the occlusal cusps of the involved mandibular molars to minimize traumatic contact. An incisional biopsy was then performed using a cold scalpel combined with CO_2_ laser, along with extraction of tooth 3.8, suspected to contribute to local trauma. Histopathological evaluation of this specimen revealed only epithelial alterations and abundant granulation tissue without evidence of neoplasia, and a definitive diagnosis could not be established at this stage (Figure 4a,b).

**Figure 5:**
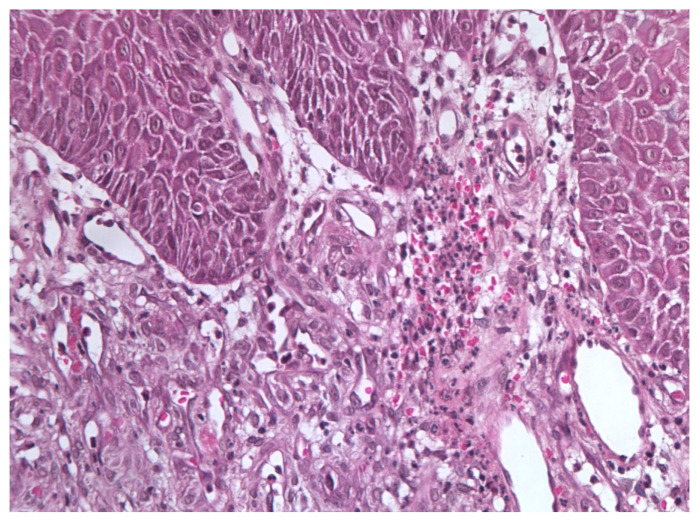
Excisional biopsy specimen (November 2015) showing endothelial cell clusters with mild atypia, numerous vascular spaces, and scattered microthrombi with hemosiderin deposits.

**Figure 6:**
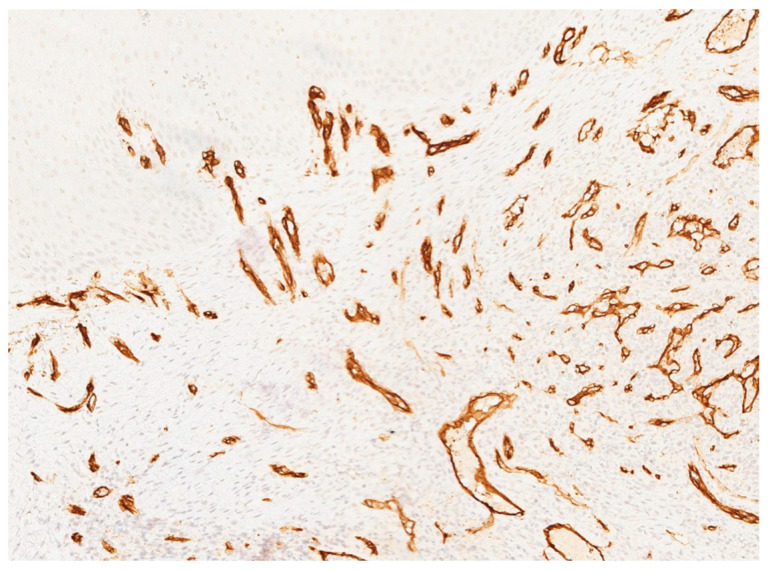
Immunohistochemical staining for CD34 showing strong and diffuse positivity of the endothelial cells lining the vascular channels, confirming the vascular nature of the lesion.

**Figure 7:**
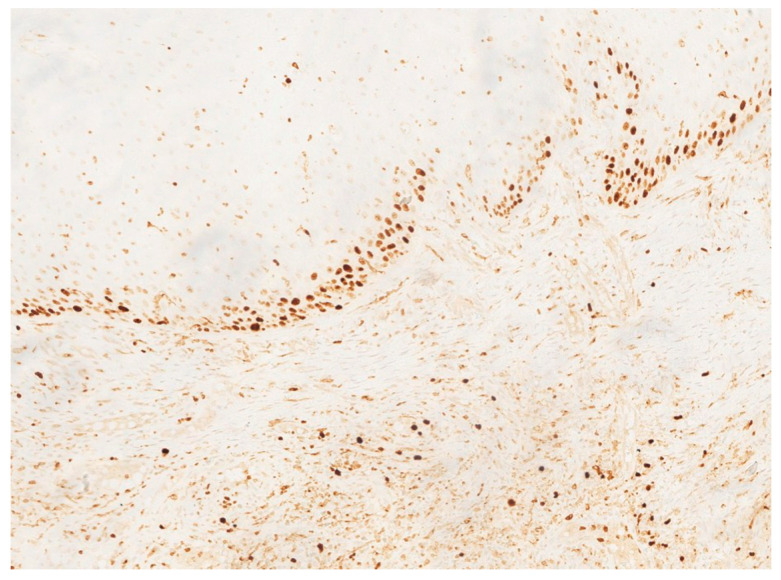
Ki-67 immunostaining demonstrating a moderate proliferative index (approximately 15%) within the endothelial component.

In November 2015, a scalpel excisional biopsy of the lesion was performed using a CO_2_ laser, together with extraction of tooth 3.8, allowing for a more representative sampling of the lesion. Histological analysis showed a partially ulcerated oral mucosa partly covered by stratified squamous epithelium with mild hyperparakeratosis, acanthosis, focal spongiosis, papillomatosis, and lymphocytic and neutrophilic exocytosis (Figure 5). Numerous capillary vascular structures positive for CD31, CD34, actin MS, and actin ML were present within the chorion, lined by swollen or spindle-shaped endothelial cells with indistinct borders, weakly eosinophilic cytoplasm, and round nuclei. These vascular structures exhibited a lobular or glomeruloid growth pattern extending into the muscle tissue, associated with an inflammatory infiltrate predominantly composed of neutrophils and fibrin thrombi within the vascular lumina. Immunohistochemistry confirmed positivity for CD31 and CD34, a Ki-67 proliferation index of 15%, and negativity for HHV-8 (Figure 6 and Figure 7). The overall morphological and immunohistochemical features were compatible with a vascular neoplasm of the oral mucosa of the kaposiform hemangioendothelioma type, as reported in the final pathology diagnosis.

The case was discussed by the Head and Neck Tumor Board, which recommended surgical excision with adequate safety margins to ensure complete removal of the lesion and prevent local recurrence. The patient was referred to the Department of Maxillofacial Surgery, where surgical resection was planned and performed according to multidisciplinary consensus. An incision was made to circumscribe the lesion of the left retromolar area with wide safety margins, including the scar from previous surgical procedures. En bloc resection of the affected mandibular segment was carried out, including the extraction of tooth 3.7. Intraoperative frozen-section histological examination revealed the presence of suspicious material along the medial resection margin; consequently, the margins were further extended (“radicalized”). A subsequent intraoperative evaluation confirmed the absence of residual disease. The surgical defect was reconstructed using a Bichat buccal fat pad flap in combination with local sliding mucosal flaps to achieve closure and restore tissue continuity.

**Figure 8:**
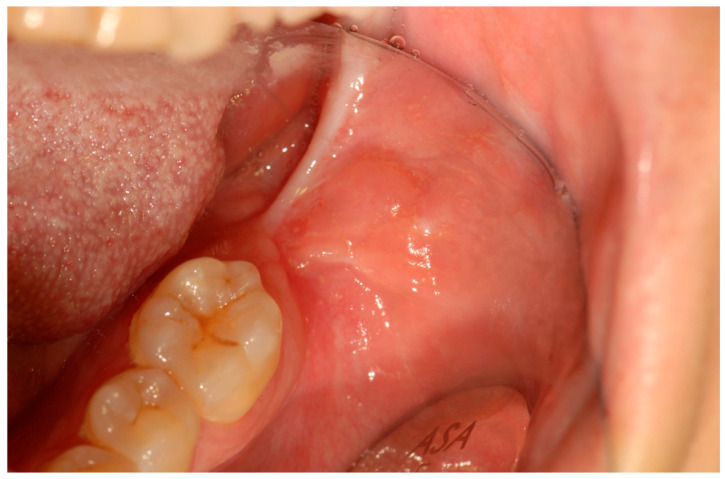
Twenty-two-month postoperative follow-up demonstrating satisfactory mucosal healing with no evidence of local recurrence.

**Figure 9:**
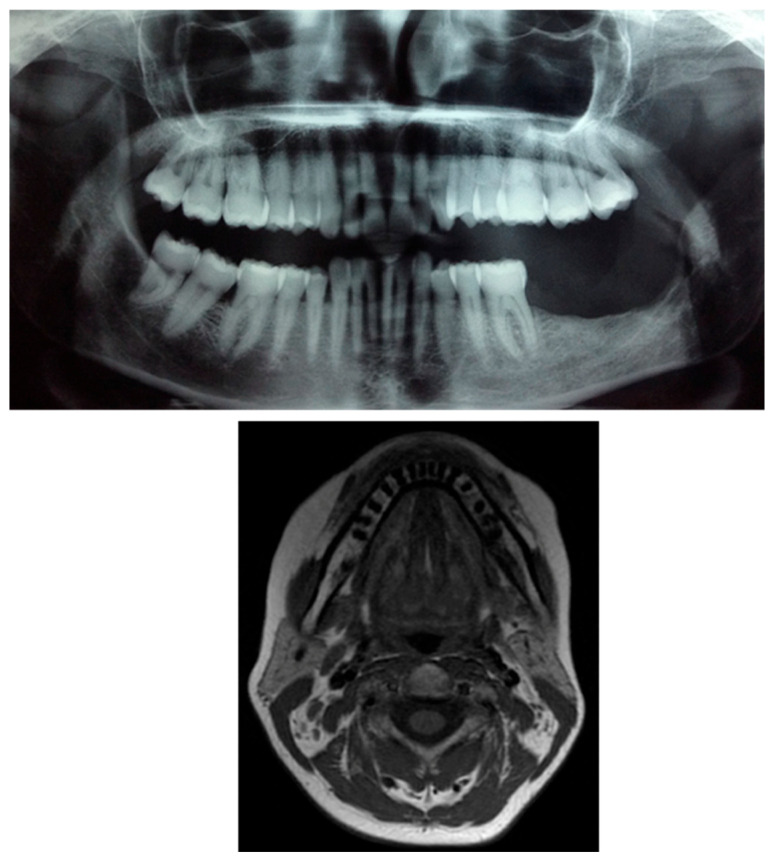
Postoperative radiographic and MRI composite images showing complete removal of the lesion and extraction of the involved teeth (3.7 and 3.8). No evidence of radiolucency or residual mass is observed in the surgical area, confirming adequate bone healing and absence of recurrence.

Postoperative management included close clinical and radiographic follow-up to monitor healing and detect possible recurrence. Unfortunately, postoperative photographic documentation was initially incomplete because the patient moved to another city shortly after surgery and continued follow-up at a different institution. However, we were able to obtain a postoperative clinical photograph taken a few months prior to her death, as well as postoperative radiographic imaging, which confirm satisfactory healing of the surgical site (Figure 8 and Figure 9). Approximately two years after surgery, the patient passed away due to cardiovascular complications unrelated to the oral neoplasm. Her death occurred after the initial diagnostic and therapeutic course, limiting further evaluation of the lesion’s long-term outcome and response to treatment.

## 3. Discussion

The term “kaposiform hemangioendothelioma” was introduced by Zukerberg et al. in 1993 [7] to describe histological aspects of this tumor that are similar to those present in cases of capillary hemangioma and Kaposi’s sarcoma.

Zhou et al. identified a somatic translocation involving chromosomes 13 and 16, specifically at loci 13q14 and 16p13.3, observed in 10% of metaphase cells from KHE lesions, along with a population of cells exhibiting a normal karyotype. Nonetheless, while GNAQ mutations have been reported in KHE, their exact contribution to tumor development remains unclear, and it is still debated whether they represent a primary driver or a secondary event [17].

KHE exhibits a wide spectrum of clinical presentations, ranging from superficial skin lesions with various morphologies to deep-seated tumors lacking cutaneous signs. Typically, it appears as a solitary soft tissue mass accompanied by skin alterations, which may include erythematous papules, plaques, nodules, or a firm, violaceous tumor. Nonetheless, approximately 12% of cases occur without any visible skin involvement [18].

Along with other vascular tumors, kaposiform hemangioendothelioma (KHE) has shown ectopic overexpression of the human Prox1 gene, a nuclear transcription factor associated with the lymphatic endothelium. In the study conducted by Borst et al. in 2024 [19], this specific Prox1-induced phenotype led to an in-depth analysis of the immunohistochemical staining pattern of Prox1, podoplanin (D2-40), LYVE-1, and Prox1/CD34, as well as dual immunofluorescent staining for LYVE-1/CD31, in KHE and tufted angioma (TA) samples. Comparison with other pediatric vascular tumors revealed that the neoplastic cells of KHE expressed Prox1+, CD31+, and CD34+ [19].

Complications in individuals with KHE are frequent. The seriousness of these complications largely depends on the patient’s age, the dimensions of the lesion, its site, its spread into deep tissues and vital organs, and any related blood disorders. It is advisable for healthcare providers to stay alert to possible complications and the risk factors that might signal future issues.

Complications like KMS manifest with an estimated incidence ranging from 42% to 71%. Thrombocytopenia in the context of KMS is often profound, with median platelet levels around 21 × 10^9^/L at diagnosis. KHE lesions linked to KMS tend to enlarge progressively and may present with purpuric discoloration. The condition can lead to significant pain and is frequently complicated by secondary bleeding [20].

According to recent studies, systemic treatments such as sirolimus, a mammalian target of rapamycin (mTOR) inhibitors, have shown efficacy in controlling disease progression in cases of unresectable or recurrent KHE. Corticosteroids and vincristine have also been proposed as therapeutic options in selected cases. However, in our patient, due to the complete excision of the lesion and the absence of KMS, additional systemic therapy was not deemed necessary at this stage.

There are about 50 cases of KHE described in the head and neck region and, of these, cases in the oral cavity are extremely rare. Among the few intraoral cases reported, all patients were male, their ages ranged from 2 to 21 years, and the lesions ranged from 1 to 3 cm in size. Our patient is a 39-year-old adult woman, and the tumor was located in the posterior mandible, presenting a different location from other previously reported intraoral cases. It is likely that KHE can occur in any area of the soft tissues in the mouth. Clinically, KHE can resemble reactive or benign vascular entities such as pyogenic granuloma or infantile hemangioma, underscoring the importance of histopathological examination, ideally supported by immunohistochemistry, for an accurate diagnosis [13].

In this instance, the lesion appeared as an exophytic growth on the alveolar ridge, accompanied by an osteolytic lesion in the adjacent alveolar bone. From a histological standpoint, KHE displays nodular arrangements of ovoid endothelial cells and vascular elements, often showing glomeruloid endothelial proliferation, ovoid cellular areas, and regions resembling lymphangiomatosis [21].

It is well known that systemic lupus erythematosus (SLE) can cause vascular alterations in the oral cavity, often attributed to chronic inflammatory phenomena, immune-mediated vasculopathies, or microangiopathy related to the underlying disease. However, oral manifestations of SLE typically present as ulcerative lesions, erythematous areas, or white striations resembling lichen planus, rather than as proliferative masses with a histological pattern similar to that of KHE. In our case, histopathology revealed the presence of spindle cell clusters and irregular vascular spaces, a finding inconsistent with vasculopathic lesions associated with SLE. Furthermore, the absence of significant perivascular lymphoplasmacytic inflammation and fibrinoid necrosis of vessel walls makes the hypothesis of an SLE-induced vasculitic alteration unlikely. Similarly, Gao and Qin (2021) [22] reported a case of a giant hemangioma in a patient with discoid lupus erythematosus, suggesting that the coexistence of autoimmune diseases and vascular proliferations does not exclude the diagnosis of a primary vascular neoplasm. This supports our interpretation, indicating that the lesion described in our study is more consistent with KHE than an atypical manifestation of SLE [22]. This locally aggressive tumor rarely involves the head and neck region and is very rarely found in adult subjects. DeFatta et al. reported a case of KHE in 2005 in a three-year-old male localized in the left hard and soft palate. The patient was completely asymptomatic, with no pain, dysphagia, odynophagia, or difficulties in breathing and speaking. The lesion was subjected to excisional biopsy, and the histopathology examination described the presence of endothelial cells with abundant eosinophilic cytoplasm, round and oval blood vessels, and spindle neoplastic endothelial cells that formed elongated vascular spaces [23].

In 2011 Rekhi et al. documented a case of KHE in the right tonsil of a two-year-old child—fortunately not associated with KMS—who presented with a clinical history of episodes of tonsillitis and ipsilateral neck swelling from birth. During one of the clinical episodes, the patient experienced acute dysphagia and dyspnea and was radiologically diagnosed with a peritonsillar abscess. Review of the histopathology slides revealed a “kaposiform” pattern featuring slit-like, crescentic capillaries with platelet thrombi, eosinophilic bodies, and prominent areas of lymphangiomatosis, which helped distinguish this case from juvenile hemangioma [24].

In 2016 Vashi et al. described the case of a 38-year-old man with a mass on the left lower surface of his tongue, which increased in size over a six-month period, with mild but asymptomatic traumatic bleeding episodes [25]. Examination of the histological samples obtained by means of an incisional biopsy led to a diagnosis of KHE and the lesion was subsequently excised [25]. In 2021 Lima Morais et al. described the case of a 10-year-old patient with a soft tissue swelling about 2 cm in diameter in the right posterior mandible [26]. A histopathology examination revealed vascular proliferation permeated by inflammatory cells and fascicles of spindle-to-ovoid cells, similar to Kaposi’s sarcoma, and the patient had to undergo extraction of the teeth involved [26]. As KHE is a very uncommon tumor, knowledge about how to treat it is not widely disseminated. Treatment options depend upon the size of the neoplasia, its surgical accessibility, and whether KMS is present. The treatment of choice, whenever possible, is most definitely surgical excision [27].

Furthermore, in the absence of any medical contraindications or a history of drug allergy, sensitivity, or reaction to vasoconstrictors (such as epinephrine), incorporating a vasoconstrictor facilitates improved hemostasis and extends the duration of anesthesia. Regardless of the method employed for local anesthetic delivery, it is essential to aspirate before injection in order to avoid inadvertent intravascular administration of both the anesthetic and vasoconstrictor. Additionally, to maximize patient comfort during the biopsy, we recommend applying a topical anesthetic with a cotton swab for one minute before puncturing the mucosa with the needle.

An incisional biopsy is recommended when sampling sizeable lesions and those suspected of being malignant. Orosco et al. conducted a comprehensive study of positive surgical margins in the ten most common solid tumors, using data from the US National Cancer Database, and identified oral cancer as the tumor most frequently exhibiting positive margins in both sexes [28]. Based on this case and the current literature, we recommend that clinicians consider KHE in the differential diagnosis of vascular lesions in the oral cavity, even in adult patients, and adopt a multidisciplinary approach to ensure accurate diagnosis and appropriate management.

A limitation of this case report is the lack of longitudinal follow-up, as the patient died from concomitant cardiovascular disease unrelated to the vascular lesion. Although this precluded extended monitoring, the clinical and histopathological findings remain relevant for highlighting the rare intraoral presentation and diagnostic challenges associated with KHE. This case also underscores the importance of timely and accurate diagnosis, as delays in recognizing rare conditions can significantly affect patient management and outcomes, as reported in hereditary dental disorders such as Type I dentin dysplasia [29].

Additionally, patient perception of the oral cavity and effective communication with the clinician are essential elements in clinical management. Innovative strategies, such as the use of clear aligners combined with smart eye-tracking technology, have demonstrated how advanced tools can enhance visualization and understanding of the oral cavity, improving communication and supporting more accurate clinical decision-making [30].

## 4. Conclusions

The diagnosis of kaposiform hemangioendothelioma remains challenging due to its rarity, particularly in the oral cavity and in adult patients. To our knowledge, no previous cases have been reported in adult women with this localization, which further complicates the diagnostic process and increases the risk of misdiagnosis or delayed treatment. Prompt recognition is essential, as the tumor’s aggressive biological behavior can lead to severe complications if left untreated.

Management requires a multidisciplinary approach supported by clinical, radiological, histological, and immunohistochemical evaluation. Careful morphological and molecular characterization is critical to differentiate KHE from other vascular lesions, such as cavernous hemangioma or other endothelial neoplasms. Early and appropriate treatment can significantly improve outcomes and prevent recurrence.

Although surgical excision remains the preferred therapeutic option when feasible, alternative treatments such as chemotherapy or targeted therapies may be considered based on the tumor’s extent and location, as well as the patient’s overall condition. A personalized approach is therefore fundamental to optimizing outcomes.

In conclusion, while KHE is an uncommon and diagnostically challenging tumor, accurate identification and timely intervention are essential for effective management and improved prognosis.

## Data Availability

No new data were created or analyzed in this study.

## Abbreviations

The following abbreviations are used in this manuscript:

KHE	Kaposiform Hemangioendothelioma
IH	Juvenile Hemangioendothelioma
KMP	Kasabach–Merritt Phenomenon
KMS	Kasabach–Merritt Syndrome

## References

[1] Croteau S.E., Liang M.G., Kozakewich H.P., Alomari A.I., Fishman S.J., Mulliken J.B., Trenor C.C. (2013). Kaposiform hemangioendothelioma: Atypical features and risks of Kasabach-Merritt phenomenon in 107 referrals. J. Pediatr..

[2] Deraedt K., Vander Poorten V., Van Geet C., Renard M., De Wever I., Sciot R. (2006). Multifocal kaposiform haemangioendothelioma. Virchows Arch..

[3] Chung M.T., Chen C.H., Chiu C.H., Yang C.P., Hsueh C., Jaing T.H. (2003). Successful nonoperative therapy for Kaposiform hemangioendothelioma involving the neck: Report of 1 case. Otolaryngol. Head Neck Surg..

[4] Tello M.A., Shields G., Gadre S.A., Ryan M. (2004). Pathology quiz case 2. Diagnosis: Kaposiform hemangioendothelioma (KHE). Arch. Otolaryngol. Head Neck Surg..

[5] Lyons L.L., North P.E., Mac-Moune Lai F., Stoler M.H., Folpe A.L., Weiss S.W. (2004). Kaposiform hemangioendothelioma: A study of 33 cases emphasizing its pathologic, immunophenotypic, and biologic uniqueness from juvenile hemangioma. Am. J. Surg. Pathol..

[6] Drolet B.A., Trenor C.C., Brandão L.R., Chiu Y.E., Chun R.H., Dasgupta R., Garzon M.C., Hammill A.M., Johnson C.M., Tlougan B. (2013). Consensus-derived practice standards plan for complicated kaposiform hemangioendothelioma. J. Pediatr..

[7] Zukerberg L.R., Nickoloff B.J., Weiss S.W. (1993). Kaposiform hemangioendothelioma of infancy and childhood. An aggressive neoplasm associated with Kasabach-Merritt syndrome and lymphangiomatosis. Am. J. Surg. Pathol..

[8] Arai E., Kuramochi A., Tsuchida T., Tsuneyoshi M., Kage M., Fukunaga M., Ito T., Tada T., Izumi M., Shimizu K. (2006). Usefulness of D2-40 immunohistochemistry for differentiation between kaposiform hemangioendothelioma and tufted angioma. J. Cutan. Pathol..

[9] Pyakurel P., Pak F., Mwakigonja A.R., Kaaya E., Heiden T., Biberfeld P. (2006). Lymphatic and vascular origin of Kaposi’s sarcoma spindle cells during tumor development. Int. J. Cancer.

[10] Ji Y., Yang K., Peng S., Chen S., Xiang B., Xu Z., Li Y., Wang Q., Wang C., Xia C. (2018). Kaposiform haemangioendothelioma: Clinical features, complications and risk factors for Kasabach-Merritt phenomenon. Br. J. Dermatol..

[11] Adams D.M., Brandão L.R., Peterman C.M., Gupta A., Patel M., Fishman S., Trenor C.C. (2018). Vascular anomaly cases for the pediatric hematologist oncologists—An interdisciplinary review. Pediatr. Blood Cancer.

[12] Mahajan P., Margolin J., Iacobas I. (2017). Kasabach-Merritt Phenomenon: Classic Presentation and Management Options. Clin. Med. Insights Blood Disord..

[13] Birchler M.T., Schmid S., Holzmann D., Stallmach T., Gysin C. (2006). Kaposiform hemangioendothelioma arising in the ethmoid sinus of an 8-year-old girl with severe epistaxis. Head Neck.

[14] Hall G.W. (2001). Kasabach-Merritt syndrome: Pathogenesis and management. Br. J. Haematol..

[15] Liu X.H., Li J.Y., Qu X.H., Yan W.L., Zhang L., Yang C., Zheng J.W. (2016). Treatment of kaposiform hemangioendothelioma and tufted angioma. Int. J. Cancer.

[16] Blatt J., Stavas J., Moats-Staats B., Woosley J., Morrell D.S. (2010). Treatment of childhood kaposiform hemangioendothelioma with sirolimus. Pediatr. Blood Cancer.

[17] Zhou S., Wang L., Panossian A., Anselmo D., Wu S., Venkatramani R. (2016). Refractory Kaposiform Hemangioendothelioma associated with the chromosomal translocation t(13;16)(q14;p13.3). Pediatr. Dev. Pathol..

[18] Gruman A., Liang M.G., Mulliken J.B., Fishman S.J., Burrows P.E., Kozakewich H.P.W., Blei F., Frieden I.J. (2005). Kaposiform hemangioendothelioma without Kasabach-Merritt phenomenon. J. Am. Acad. Dermatol..

[19] Borst A.J., Eng W., Griffin M., Ricci K.W., Engel E., Adams D.M., Dayneka J., Cohen-Cutler S.J., Andreoli S.M., Wu M.D. (2024). Treatment practices and response in kaposiform hemangioendothelioma: A multicenter cohort study. Pediatr. Blood Cancer.

[20] Rodriguez V., Lee A., Witman P.M., Anderson P.A. (2009). Kasabach-Merritt Phenomenon. J. Pediatr. Hematol. Oncol..

[21] Sreenivasan B.S., Ambooken M., Radhakrishna M., Sebastian J. (2015). An intraoral epitheloid hemangioendothelioma masquerading clinically as pyogenic granuloma. Iran. J. Med. Sci..

[22] Gao W., Qin X. (2021). Cutaneous and giant hepatic haemangioma associated with Kasabach-Merritt syndrome in an adult patient with discoid lupus erythematosus. Postepy Dermatol. Alergol..

[23] DeFatta R.J., Verret D.J., Adelson R.T., Gomez A., Myers L.L. (2005). Kaposiform hemangioendothelioma: Case report and literature review. Laryngoscope.

[24] Rekhi B., Sethi S., Kulkarni S.S., Jambhekar N.A. (2011). Kaposiform hemangioendothelioma in tonsil of a child associated with cervical lymphangioma: A rare case report. World J. Surg. Oncol..

[25] Vashi P., Abboud E., Bier-Laning C., Gupta D. (2016). Adult-onset Kaposiform hemangioendothelioma of the tongue: Case report and review of the literature. Curr. Oncol..

[26] Morais T.M.L., Sánchez-Romero C., Ribeiro L., Faé D.S., Verner F.S., de Almeida O.P., de Aquino S.N. (2021). Kaposiform Hemangioendothelioma of the Oral Cavity: A Rare Tumor with an Unusual Location. Head Neck Pathol..

[27] Yang H., Wang J., Song L., Zou H. (2019). Intraosseous epithelioid haemangioendothelioma of the mandible: A case report and literature review. Medicine.

[28] Orosco R.K., Tapia V.J., Califano J.A., Clary B., Cohen E.E.W., Kane C., Lippman S.M., Messer K., Molinolo A., Murphy J.D. (2018). Positive Surgical Margins in the 10 Most Common Solid Cancers. Sci. Rep..

[29] Putrino A., Caputo M., Galeotti A., Marinelli E., Zaami S. (2023). Type I Dentin Dysplasia: The Literature Review and Case Report of a Family Affected by Misrecognition and Late Diagnosis. Medicina.

[30] Putrino A., Marinelli E., Raso M., Calace V., Zaami S. (2023). Clear Aligners and Smart Eye Tracking Technology as a New Communication Strategy between Ethical and Legal Issues. Life.

